# Supplementary figures and images for: Isolation, identification, and biological characteristics of Clostridium sartagoforme from rabbit
Source: PLoS One. 2021 Nov 15;16(11):e0259715. doi: 10.1371/journal.pone.0259715 (PMC8592454; doi:10.1371/journal.pone.0259715)

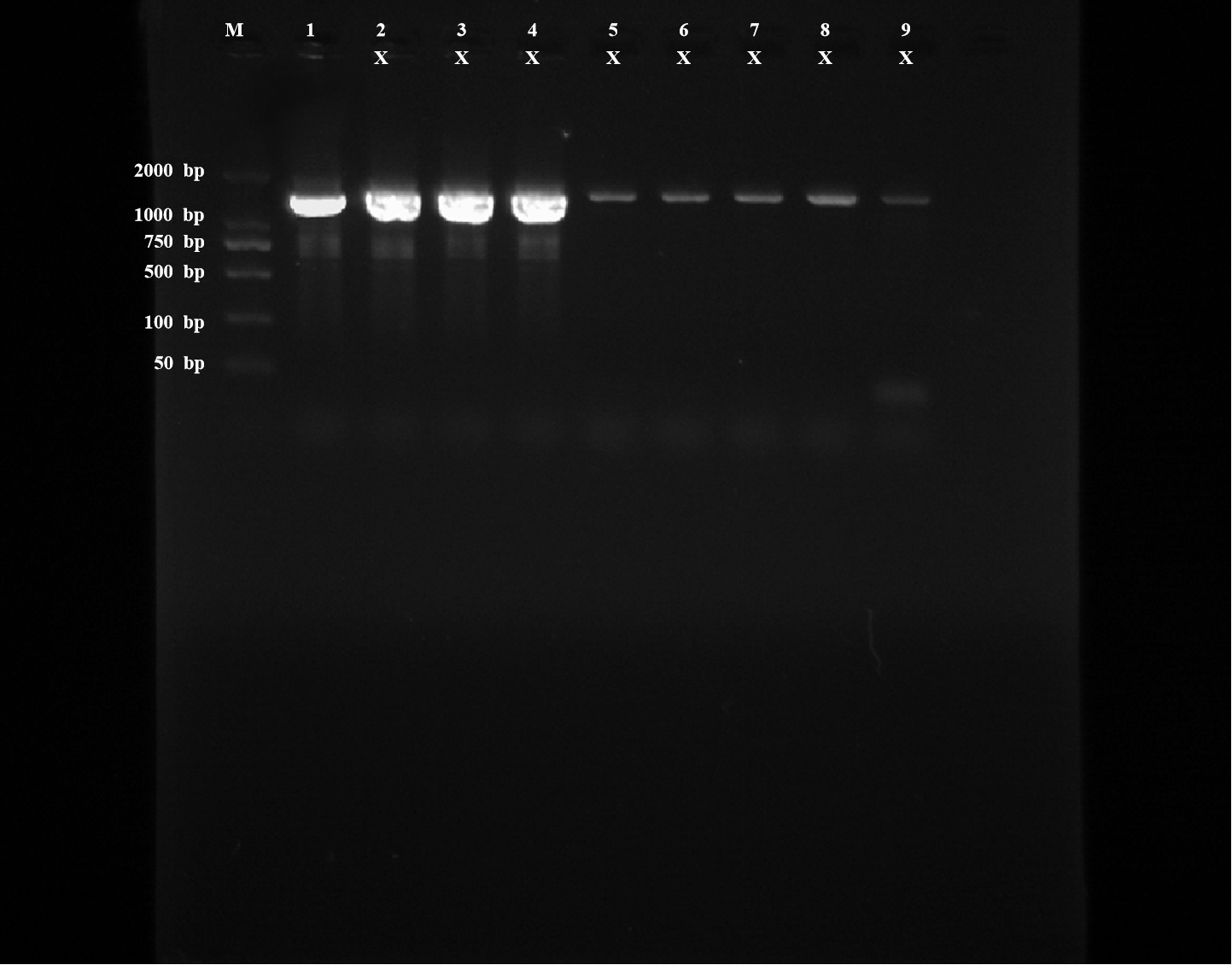

Supplement: S1 Fig — (TIF) [file pone.0259715.s002.tif]
