# Supplementary material for: Isolation, identification, and biological characteristics of Clostridium sartagoforme from rabbit
Source: PLoS One. 2021 Nov 15;16(11):e0259715. doi: 10.1371/journal.pone.0259715 (PMC8592454; doi:10.1371/journal.pone.0259715)
Supplement: S1 Raw images — (PDF) [file pone.0259715.s003.pdf]

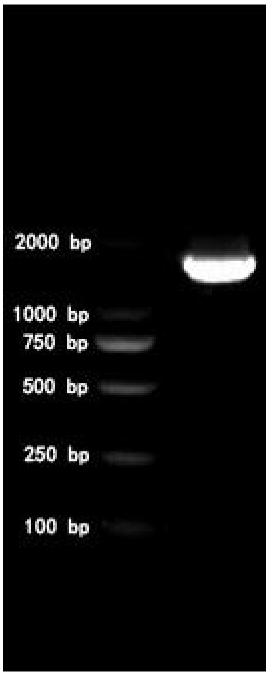

Fig 1. Agarose gel electrophoresis of *C. sartagoforme XN-T4* 16S rDNA.

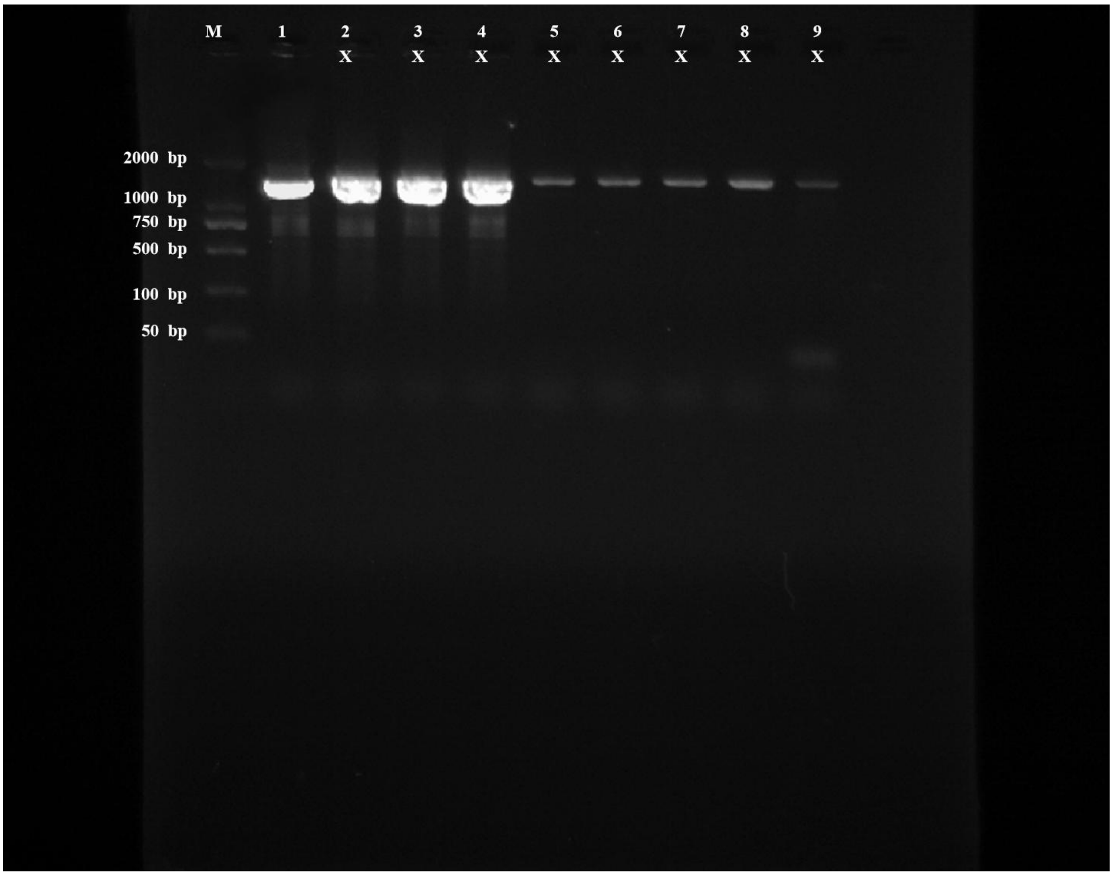

Supplemental Figure. Original blot and gel images

Notes: M Molecular weight markers

1 Clostridium sartagoforme XN-T4 （This is the blot of our target strain）

2 Clostridium tertium

3 Clostridium sordellii
4 Clostridium tertium
5 Clostridium tertium
6 Clostridium sordellii
7 Clostridium tertium
8 Clostridium tertium
9 Clostridium tertium
