# Supplementary material for: Isolation, identification, and biological characteristics of Clostridium sartagoforme from rabbit
Source: PLoS One. 2021 Nov 15;16(11):e0259715. doi: 10.1371/journal.pone.0259715 (PMC8592454; doi:10.1371/journal.pone.0259715)
Supplement: Attachment — Submitted filename: Response to reviewers-3.doc [file pone.0259715.s004.doc]

Response to reviewers-3

1. Ren Zhanjun was the specific recipient of the five fundings [ (1) Shaanxi Province Science and Technology Project (K3310216062); (2) Shaanxi Province Agricultural Science and Technology Innovation and Research Project (16NY-108); (3) Shaanxi Province Agricultural Science and Technology Innovation Transformation Project (NYKJ-2020-YL-16); (4) Yangling Demonstration Zone Industry-University-Research Collaborative Innovation Major Project (1017cxy-15); (5) Key R&D project of Shaanxi Province (2018ZDXM-NY-041)] .
2. The methods of animal experiments were accordance with the relevant regulations by the Committee on Laboratory Animals of Northwest Agriculture & Forest University (NO. 0369/2018), and the pdf of animal experiments permission had been added as “supporting information”.
