# Supplementary material for: Isolation, identification, and biological characteristics of Clostridium sartagoforme from rabbit
Source: PLoS One. 2021 Nov 15;16(11):e0259715. doi: 10.1371/journal.pone.0259715 (PMC8592454; doi:10.1371/journal.pone.0259715)
Supplement: Attachment — Submitted filename: Reviewers comments-2021,4,25.docx [file pone.0259715.s005.docx]

Reviewers' comments:

The authors revised the last version according to this reviewer's comments. However, I still have four points to be revised.

1. This manuscript reports the isolation, identification and biological characteristics of *Clostridium sartagoforme* from rabbit. However, it is not rigorous and unscientific. For using as microbial feed additive, the authors should add more research data on the probiotic effect and safety of *Clostridium sartagoforme* in vitro.

2. In Introduction, the authors propose that *Clostridium sartagoforme* had potential properties as a microbial food additives. However, following this introduction, there is little mention of this same notion. The authors should give a review of the pertinent literature, please include more details. What were the motivations and purpose for isolating *Clostridium sartagoforme*?

3. In Discussion, while the authors state in the abstract that their study provides evidence of the potential utility of *Clostridium sartagoforme* as a microbial food additive, there is essentially no discussion of the results that help support this point. Additionally, and even more critically, there is little discussion as to why *Clostridium sartagoforme* would be beneficial as a microbial food additive.

4. All taxa names (species names, genus names, and names of higher categories) should be italicized.
