# Supplementary material for: Isolation, identification, and biological characteristics of Clostridium sartagoforme from rabbit
Source: PLoS One. 2021 Nov 15;16(11):e0259715. doi: 10.1371/journal.pone.0259715 (PMC8592454; doi:10.1371/journal.pone.0259715)
Supplement: Attachment — Submitted filename: Response to Reviewers.doc [file pone.0259715.s006.doc]

Response to reviewers-4

**Abstract: It would benefit the paper if information concerning the rabbit testing of C. sartagoforme was added to the abstract**

The sentence of “Moreover, animal testing indicated that this strain has no adverse effects on the morbidity and mortality of rabbits.” has been added.

**Line 50: Please consider adding a reference for this sentence.**

**Citations has been added:**

[1] Ayyat MS, Al-Sagheer AA, Abd El-Latif KM, Khalil BA. Organic Selenium, Probiotics, and Prebiotics Effects on Growth, Blood Biochemistry, and Carcass Traits of Growing Rabbits During Summer and Winter Seasons. Biol Trace Elem Res. 2018 Nov;186(1):162-173. doi: 10.1007/s12011-018-1293-2. Epub 2018 Mar 7. PMID: 29516355.

[2]Pogány Simonová M, Chrastinová Ľ, Lauková A. Autochtonous Strain Enterococcus faecium EF2019(CCM7420), Its Bacteriocin and Their Beneficial Effects in Broiler Rabbits-A Review. Animals (Basel). 2020 Jul 14;10(7):1188. doi: 10.3390/ani10071188. PMID: 32674281; PMCID: PMC7401553.

[3]Elghandour MMY, Tan ZL, Abu Hafsa SH, Adegbeye MJ, Greiner R, Ugbogu EA, Cedillo Monroy J, Salem AZM. Saccharomyces cerevisiae as a probiotic feed additive to non and pseudo-ruminant feeding: a review. J Appl Microbiol. 2020 Mar;128(3):658-674. doi: 10.1111/jam.14416. Epub 2019 Sep 8. PMID: 31429174.

[4]Zhou Y, Ni X, Wen B, Duan L, Sun H, Yang M, Zou F, Lin Y, Liu Q, Zeng Y, Fu X, Pan K, Jing B, Wang P, Zeng D. Appropriate dose of Lactobacillus buchneri supplement improves intestinal microbiota and prevents diarrhoea in weaning Rex rabbits. Benef Microbes. 2018 Apr 25;9(3):401-416. doi: 10.3920/BM2017.0055. Epub 2018 Jan 30. PMID: 29380642.

[5]Wang J, Ni X, Wen B, Zhou Y, Liu L, Zeng Y, Zhao W, Khalique A, Wang P, Pan K, Yu Z, Jing B, Liu H, Zeng D. Bacillus strains improve growth performance via enhancing digestive function and anti-disease ability in young and weaning rex rabbits. Appl Microbiol Biotechnol. 2020 May;104(10):4493-4504. doi: 10.1007/s00253-020-10536-9. Epub 2020 Mar 19. PMID: 32193576.

[6]El-Deep MH, Dawood MAO, Assar MH, Ahamad Paray B. Aspergillus awamori positively impacts the growth performance, nutrient digestibility, antioxidative activity and immune responses of growing rabbits. Vet Med Sci. 2021 Jan;7(1):226-235. doi: 10.1002/vms3.345. Epub 2020 Sep 9. PMID: 32902158; PMCID: PMC7840208.

**Line 51: Please consider adding a reference for this statement.**

**Citations has been added:**

[7]Wang Z. Preparation of Lactic acid bacteria Preparation an its Preliminary Application in Weaned Piglets [dissertation]. Harbin: Northeast Agricultural University; 2019.

[8]Bintsis T. Lactic acid bacteria as starter cultures: An update in their metabolism and genetics. AIMS Microbiol. 2018 Dec 11;4(4):665-684. doi: 10.3934/microbiol.2018.4.665. PMID: 31294241; PMCID: PMC6613329.

**Line 54: Please consider adding a reference for this statement concerning Bacillus.**

**Citation has been added:**

[9]Hu JH. Handbook of Medication. 4th ed. China Beijing: Jundun Press; 2009. Chinese.

**Line 107: Please consider adding a reference for this statement. It may also be prudent to further explain this and the mechanism (over-decolorization) in some more detail.**

**Citation has been added:**

[12]Shen P, Chen XD. Microbiology. 8th ed. Beijing: Higher Education Press; 2016. Chinese.

**Explain:** Clostridium is generally positive for Gram staining, but the cell wall structure of the old bacteria would become loose if the culture time is too long, and the result of Gram negative may appear after staining.

**Line 112: For the sake of reproducibility, could the authors please provide some further details on the genomic DNA extraction kit that was used?**

Details on the bacteria genomic DNA extraction kit that was added：Takara Bio Inc., Shiga, Japan.

**Line 162: This difficult is difficult to read and should be rewritten (especially the end) for further clarity.**

**The methods were clarified as：**

After incubation, bacteria were washed three times with phosphate-buffered saline (PBS; pH 7.2), 10-fold serial dilutions were made, and then, colony-forming units were counted on RCM agar plates after 48 h of incubation at 37 ℃. Tolerance was expressed as a percentage of the number of colony-forming units after incubation in artificial gastric acid relative to that of the normal RCM.

**Line 172: It is unclear to me exactly how many rabbits were in each experimental group. Please consider revising this section for more clarity. A simple figure may also help this portion be more easily interpreted.**

**The methods were clarified as：**

The experiment was conducted in a completely randomized design with 3 treatments, and every treatment with 3 replicates, 18 rabbits in each replicate (162 Rex rabbits 35-day-old ) that included the following treatments.

**Line 193: Can you please describe the statistical tests that were used to compare the control and treatment groups for the trials including the rabbits?**

Mortality and diarrhea rate of rabbits were analysised by one-way analysis of variance with SPSS 22.0 software (SPSS Inc., Chicago, IL, USA).

**Table 3: C. sartagoforme should have returned a negative result for the utilization of sorbitol. Were any steps taken to control for potential contamination in the experiments?**

The bacteria were inoculated in the clean bench, and the biochemical reaction tube was liquid sealed with sterilized liquid paraffin and covered with parafilm.

**Line 282: As mentioned previously, how were significant differences between treatment groups of rabbits evaluated?**

Data of mortality and diarrhea rate have been reanalyzed, and the significant differences between treatment groups of rabbits have been marked.

**Table 7: It is curious that the diarrhea rate and mortality rate for group A are identical. Additionally, the standard error of the means appears to be very large for the diarrhea rate, this causes some concern for the conclusions that are drawn here. This concern may be alleviated once the analysis performed is described in more detail.**

Data of mortality and diarrhea rate have been reanalyzed, but the standard error of the means still large, especially the numerical value of mortality are the same in different treament, the reason is that only 1 rabbit died in each group. Raw Data of Mortality and Diarrhea Rate as follow:

Raw Data of Mortality and Diarrhea Rate

| Total number of rabbits | Group |  | Mortality | Diarrhea rate |
| --- | --- | --- | --- | --- |
| 162 | A | Replicate 1 | 5.56 | 0.79 |
|  |  | Replicate 2 | 0 | 0.79 |
|  |  | Replicate 3 | 0 | 0.48 |
|  | B | Replicate 1 | 5.56 | 0.95 |
|  |  | Replicate 2 | 0 | 0.79 |
|  |  | Replicate 3 | 0 | 0.79 |
|  | C | Replicate 1 | 0 | 1.11 |
|  |  | Replicate 2 | 5.56 | 0.63 |
|  |  | Replicate 3 | 0 | 0.49 |

Notes: A, B and C respectively represent the amount of bacteria added according to the body weight of Rex rabbits: 10^8^ CFU/Kg, 10^6^ CFU/Kg and medium mixture.

**Line 294: It would be beneficial to add a citation for this sentence.**

**Citation has been added:**

[18]Sun XM. Effect of L-arginine and N-carbamylgluatmate on small intestinal villi development and cecal microbiota composition of young rabbits [dissertation]. Changchun: JiLin University; 2019.

**Line 303: The use of the term “molecular clock” is interesting here. I’m not sure that this is the best term to describe this. It seems that “molecular barcode” or “molecular identifier” would be some potential alternatives.**

Molecular clock had been changed as molecular identifier.

**Line 314: It would be beneficial for a citation to be added here.**

**Citation has been added:**

[20]Chen JD, Huang QY. Animal husbandry microbiology. 6th ed. China Beijing: Agriculture Press; 2017. Chinese.

**Lines 330-334: The manuscript would be improved if some references were added for each of these statements.**

**Citations has been added:**

**Line 330:**

[24]Ouyang WQ. Animal Physiology. Beijing: Science Press; 2021. Chinese.

**Line 331:**

[25]Guo ZQ, Wang B, Kuang LD, Yang R, Li CY, Zheng J. Gastrointestinal development and cecal cellulase activity in Qixing and New Zealand rabbits. Chinese Journal of Animal Science. 2021,57(03):130-133. Chinese

**Line 332:**

[26]Simonová MP, Chrastinová L, Kandricáková A, Kubašová I, Formelová Z, Chrenková M, Miltko R, Belzecki G, Strompfová V, Lauková A. Enterocin M and Sage Supplementation in Post-weaning Rabbits: Effects on Growth Performance, Caecal Microbiota, Fermentation and Enzymatic Activity. Probiotics Antimicrob Proteins. 2020 Jun;12(2):732-739. doi: 10.1007/s12602-019-09584-z. PMID: 31414382.

**Line 334:**

[27]Gu ZL, Qing YH, Ren KL. China rabbit Science. China Beijing: China Agriculture Press; 2013. Chinese.

**Line 334: There appears to be some typos and confusion in this sentence.**

**This sentence has been changed as:**

The transparent circle on the CMC-Congo Red plate indicates that the bacterium can decompose cellulose, it means that *C. sartagoforme XN-T4* has the potential to improve the digestibility of cellulose for herbivores as a microbial feed additive.

**Line 341: It would be beneficial for a citation to be added here.**

**Citation has been added:**

[28]Dong XZ, Cai MY. System Identification Manual of Common Bacteria. Beijing: Science Press; 2001. Chinese.

**Line 346: It would be beneficial for a citation to be added here.**

**Citation has been added:**

[29]Yu LJ. Principles and Technology of Fermentation Engineering. Beijing: Higher Education Press; 2021. Chinese.

**Line 355: It would be beneficial for a citation to be added here.**

**Citation has been added:**

[29]Yu LJ. Principles and Technology of Fermentation Engineering. Beijing: Higher Education Press; 2021. Chinese.

**Line 371-374: Both of these references are for studies involving mice. Do these statements hold true for rabbits as well?**

These studies are mainly on mice, and there are no rabbits, so I have to refer to mice here.

**Line 385: This concluding sentence seems weirdly informal and would benefit from some rewriting.**

**The conclusion rewrited as:**

A strain of *Clostridium sartagoforme XN-T4* was isolated from Hyla rabbit feces by RCM medium. Through colony characteristics and cell morphology, combined with physiological and biochemical methods and PCR identification. The strain has good stress resistance, can decompose cellulose, and has no obvious toxic effect on rabbits. It may be a candidate for microbial additives to improve the digestibility of cellulose in rabbits. Futher more, for its secondary metabolite composition, safety and in vitro and in vivo metabolic characteristics still need experimental research.

**Reviewers' comments:**

**1.This manuscript reports the isolation, identification and biological characteristics of Clostridium sartagoforme from rabbit. However, it is not rigorous and unscientific. For using as microbial feed additive, the authors should add more research data on the probiotic effect and safety of Clostridium sartagoforme in vitro.**

Dear reviewer, I would like to further the research on this strain. However, as I have graduated, I have no conditions and equipment to invest in this field. At present, I am applying for a doctor's degree, after getting the offer, I plan to continue the research on the probiotic effect and safety of *Clostridium sartagoforme* in vitro.

**2. In Introduction, the authors propose that Clostridium sartagoforme had potential properties as a microbial food additives. However, following this introduction, there is little mention of this same notion. The authors should give a review of the pertinent literature, please include more details. What were the motivations and purpose for isolating Clostridium sartagoforme?**

The motivations and purpose were to isolate a strain suitable for rabbits, which can resist the effect of digestive juice and improve the digestibility of cellulose in rabbits. By adding this strain, the economic benefit of rabbit breeding can be improved.

**3. In Discussion, while the authors state in the abstract that their study provides evidence of the potential utility of Clostridium sartagoforme as a microbial food additive, there is essentially no discussion of the results that help support this point. Additionally, and even more critically, there is little discussion as to why Clostridium sartagoforme would be beneficial as a microbial food additive.**

The reason for *Clostridium sartagoforme* would be beneficial as a microbial food additive as fellow:

The strain has no obvious toxic effect on rabbits, to a certain extent, it indicates that the bacterium is not a pathogen.

The strain has good stress resistance which can resist the effect of digestive juice, this means that the bacteria may reach the rabbit's cecum to play a role.

The strain can decompose cellulose, it is possible that the strain can improve feed utilization by decomposing cellulose.

**4. All taxa names (species names, genus names, and names of higher categories) should be italicized.**

Taxa names had been italicized in the article.
