# Supplementary material for: Isolation, identification, and biological characteristics of Clostridium sartagoforme from rabbit
Source: PLoS One. 2021 Nov 15;16(11):e0259715. doi: 10.1371/journal.pone.0259715 (PMC8592454; doi:10.1371/journal.pone.0259715)
Supplement: Attachment — Submitted filename: Reviewers comments-2021,7,27.docx [file pone.0259715.s007.docx]

Reviewers' comments:

The authors revised the last version according to reviewers’ comments. However, I still have some points to be revised.

1. In the last review, I commented “the authors should add more research data on the probiotic effect and safety of *Clostridium sartagoforme* in vitro”. The author did not realize the importance of its safety and effectiveness. In any case, as microbial a feed additive, the safety of probiotics is very important. If there is no guarantee that *Clostridium sartagoforme* is safe, all subsequent studies will be futile. Therefore, some basic safety tests such as antibiotic susceptibility test, hemolytic test, and toxicity test are necessary.

2. In the last review, I commented “The authors should give a review of the pertinent literature, please include more details.” in the introduction. The author did not make changes in accordance with the comments, and the explanation given was unreasonable. The introduction should provide with sufficient background information to introduce the reader into the reported study. If the author is not clear about the relevant research progress of *Clostridium sartagoforme*, how does the author think that it can be used as a microbial food additive? A review of the pertinent literature is necessary and it can well reflect the authors' motivations and purpose for isolating *Clostridium sartagoforme*.

3. Regarding the authors’ response to the reviewers-4 comments, there are a few points, and I would like to give my own comments.

(1) Whether there are other more reasonable explanations for changing from Gram-positive to Gram-negative of *Clostridium sartagoforme*.

(2) In Line 162, reviewers-4 commented “This difficult is difficult to read and should be rewritten (especially the end) for further clarity”, the authors clarified the methods, but there are still some details that you need to add. After 10-fold serial dilutions, how many dilutions should be taken, whether it is spread or spotted, and how is the colony-forming units calculated?

(3) In Line 172, reviewers-4 commented “It is unclear to me exactly how many rabbits were in each experimental group.”, but there are still some details that you need to add. Are female rabbits or male rabbits used in the experiment, and what is the ratio?

(4) The results of diarrhea rate and mortality are quite different before and after, please confirm the accuracy and authenticity of the data. The formula for calculating the diarrhea rate may be wrong, please confirm.

(5) In Line 371-374, reviewers-4 commented “Both of these references are for studies involving mice. Do these statements hold true for rabbits as well?”, the authors responded “These studies are mainly on mice”. Please change references for studies related to rabbits.

(6) Reviewers-4 suggested that the author added a lot of references, but most of the newly added references are in Chinese. Please replace some of the references with English references.
