# Supplementary material for: Isolation, identification, and biological characteristics of Clostridium sartagoforme from rabbit
Source: PLoS One. 2021 Nov 15;16(11):e0259715. doi: 10.1371/journal.pone.0259715 (PMC8592454; doi:10.1371/journal.pone.0259715)
Supplement: Attachment — Submitted filename: Response to Reviewer #5.docx [file pone.0259715.s008.docx]

Response to Reviewer#5:

**Point 1:** In the last review, I commented “the authors should add more research data on the probiotic effect and safety of *Clostridium sartagoforme* in vitro”. The author did not realize the importance of its safety and effectiveness. In any case, as microbial a feed additive, the safety of probiotics is very important. If there is no guarantee that *Clostridium sartagoforme* is safe, all subsequent studies will be futile. Therefore, some basic safety tests such as antibiotic susceptibility test, hemolytic test, and toxicity test are necessary.

**Response 1:** Thank you for your instructive suggestions. The studies of diarrhea rate and mortality rate on rabbits were used to demonstrate the safety of this strain, and further experiments such as cellulase production, components of secondary metabolites, antibiotic susceptibility test, hemolytic test, toxicity test, blood biochemical inedxes and organ coefficients of experimental animals will be carry out.

**Point 2:** In the last review, I commented “The authors should give a review of the pertinent literature, please include more details.” in the introduction. The author did not make changes in accordance with the comments, and the explanation given was unreasonable. The introduction should provide with sufficient background information to introduce the reader into the reported study. If the author is not clear about the relevant research progress of *Clostridium sartagoforme*, how does the author think that it can be used as a microbial food additive? A review of the pertinent literature is necessary and it can well reflect the authors' motivations and purpose for isolating *Clostridium sartagoforme*.

**Response 2:** Thank you for your careful reading of our manuscript. The introduction has been rewrote as follow:

Rabbits are herbivores, there are abundant cellulose degrading bacteria resources in rabbit intestinal tract, because they rely on the microorganisms in the caecum to digest cellulose but not produce cellulase by themselves. In addition, cellulose degrading bacteria isolated from animals have natural advantages in the utilization of crude fiber feed. Most of them are inherent bacteria in the intestinal tract, which are easier to adapt to the intestinal environment of the animal and play a role.

*Clostridium sartagoforme* is an anaerobic spore producing bacterium, which determines that the bacterium has a natural advantage in resisting the effects of digestive juice and adapting to the anaerobic environment of the digestive tract. Researches have shown that the strain can decompose microcrystalline cellulose, carboxymethyl cellulose and chitin, and its metabolites include formic acid, acetic acid, propionic acid, lactic acid, butyric acid, isovaleric acid and hydrogen. Simůnek J et al. isolated *C. sartagoforme* from feces of horse, and this strain has high exocellular activity of N-Acetylglucosaminidase. Zhang JN isolated *Clostridium sartagoforme FZ11* from cow dung compost, using urea as a nitrogen source and raw corn stalk as a substrate, confirming that this strain could decompose cellulose and have good hydrogen production characteristics. In Nathani Neelam M and coleague’s research, *Clostridium sartagoforme AAU1* was isolated from Surti buffalo rumen liquor, and they pointed out that VFAs produced by the bacteria play an important role in animal health maintenance through genomic analysis. At present, the research about *C. sartagoforme* isolated from digestive tract or feces of rabbits has not been reported, and there is little information on the biological characteristics of this strain. In this experiment, *C. sartagoforme* was isolated and identified from rabbit feces, and its physiological, biochemical and stress resistance characteristics were preliminarily studied, which help to provide reference for the further development of rabbit microbial additives.

**Point 3-(1):** Whether there are other more reasonable explanations for changing from Gram-positive to Gram-negative of *Clostridium sartagoforme*.

**Response 3-(1):** Thank you for your careful reading of our manuscript. For this phenomenon, only this point of view is currently being seen.

**Point 3-(2):** In Line 162, reviewers-4 commented “This difficult is difficult to read and should be rewritten (especially the end) for further clarity”, the authors clarified the methods, but there are still some details that you need to add. After 10-fold serial dilutions, how many dilutions should be taken, whether it is spread or spotted, and how is the colony-forming units calculated?

**Response 3-(2):** I'm sorry for the article writing problem. Samples were diluted up to 10^-8^, 50 μL of the diluted cultures was spread on RCM agar plates and incubated for 48 h at 37 ℃, 6 replicates for each sample, followed by colony counting. The survival rate (%) in gastric acid was calculated with the mean of colony counts of the plates after treatment with respect to their mean before treatment. The results were presented in mean number of surviving bacteria ± standard deviation.

**Point 3-(3):** In Line 172, reviewers-4 commented “It is unclear to me exactly how many rabbits were in each experimental group.”, but there are still some details that you need to add. Are female rabbits or male rabbits used in the experiment, and what is the ratio?

**Response 3-(3):** I apologize for the ambiguity in the description. A total of 162 35-day-old Rex rabbits (half males and half females) were randomly divided into 3 groups with 9 replicates per group and 6 rabbits per replicate.

**Point 3-(4):** The results of diarrhea rate and mortality are quite different before and after, please confirm the accuracy and authenticity of the data. The formula for calculating the diarrhea rate may be wrong, please confirm.

**Response 3-(4):** Thank you for your valuable advice. The diarrhea rate has been checked to be correct, but the formula for diarrhea rate is indeed not accurate enough, it has been changed as:

$$\text{Diarrhea rate}=\frac{\text{Cumulative days of diarrhea rabbits in each replicate}}{\text{Number of rabbits in each replicate× Feeding days}}\times100\text{\%}$$

**Point 3-(5):** In Line 371-374, reviewers-4 commented “Both of these references are for studies involving mice. Do these statements hold true for rabbits as well?”, the authors responded “These studies are mainly on mice”. Please change references for studies related to rabbits.

**Response 3-(5):** Thank you for your careful work. The reference about “gastric juice and pH in stomach” had been changed to those researches on rabbits. The results of this study are indeed very different in rabbits and mice. I am ashamed of the previous erroneous quotation of references and sincerely thank you for your suggestions.

**Point 3-(6):** Reviewers-4 suggested that the author added a lot of references, but most of the newly added references are in Chinese. Please replace some of the references with English references.

**Response 3-(6):** According to the reviewer’s comments, most of the references in Chinese had been changed to English references.
