# Supplementary material for: Isolation, identification, and biological characteristics of Clostridium sartagoforme from rabbit
Source: PLoS One. 2021 Nov 15;16(11):e0259715. doi: 10.1371/journal.pone.0259715 (PMC8592454; doi:10.1371/journal.pone.0259715)
Supplement: Attachment — Submitted filename: Reviewers comments-2021,10,25.docx [file pone.0259715.s009.docx]

Reviewers' comments:

The authors have revised the version according to reviewers’ comments. I recommend publishing the manuscript. However, the format of the manuscript needs to be carefully modified in accordance with the standards required by the journal.
